# Naturally Occurring Fc-Dependent Antibody From HIV-Seronegative Individuals Promotes HIV-Induced IFN-α Production

**DOI:** 10.1038/srep37493

**Published:** 2016-11-24

**Authors:** Thomas Lum, Jon A. Green

**Affiliations:** 1 East Bay Institute for Research and Education, Mather, California, USA; 2 Associate Chief of Staff Department of Veterans Affairs Medical Center, Sacramento, California, USA.

## Abstract

A majority of adults without HIV infection and with a low risk of HIV-exposure have plasma IgG antibodies that enhance the rate and magnitude of HIV-induced interferon alpha (IFN-α) production. Fc-dependent IgG-HIV complexes induce IFN-α rapidly and in high titers in response to HIV concentrations that are too low to otherwise stimulate an effective IFN-α response. IFN-α promoting antibody (IPA) counters HIV-specific inhibition of IFN-α production, and compensates for the inherent delay in IFN-α production common to HIV infection and other viruses. Naturally occurring IPA has the potential to initiate a potent IFN-α response early in the course of HIV mucosal invasion in time to terminate infection prior to the creation of a pool of persistently infected cells. The current study adds IPA as a mediator of an Fc-dependent antiviral state capable of preventing HIV infection.

*Human immunodeficiency virus* (HIV) infection is relatively difficult to acquire, and large numbers of unprotected heterosexual exposures are needed to produce a single infection^1^,^2^,^3^. Successful transmission initiated by a single transmitted founder virus occurs most commonly at a mucosal surface^4^,^5^,^6^. Reports of IFN-α resistant founder virus suggests that IFN-α can be protective in cases where infection is aborted^5^,^7^,^8^,^9^. However, there are limitations in postulating a definitive role for HIV-induced interferon in preventing infection. Although IFN-α is the central mediator of the innate antiviral immune response, its efficacy is limited by slow production and low initial titers^10^,^11^,^12^. Typically, multiple cycles of virus replication are needed to create virus concentrations capable of inducing IFN-α production, but only a few cycles of replication are needed for HIV to establish a pool of permanently infected cells^13^. In addition HIV further delays the onset and magnitude of IFN-α production^9^,^14^,^15^,^16^. In order to terminate HIV replication IFN-α would require the participation of as yet unidentified host factors capable of augmenting its production.

Previously, we have shown that serum immunoglobulin G (IgG) from individuals with advanced HIV infection markedly enhanced HIV-induced IFN-α production *in vitro*^17^. IgG capable of intensifying the IFN-α response has also been demonstrated for other viruses to which humans and animals have antibodies as a result of prior infection, immunization or environmental exposure^17^,^18^,^19^,^20^,^21^,^22^,^23^. However, prior viral exposure is not essential, and a majority of adults without identifiable *vesicular stomatitis virus* (VSV) exposure have serum IgG that enhances the rate and magnitude of VSV induced IFN-α production^24^. Regardless of its origins antibody that enhances virus-induced IFN-α production combines the antigenic specificity of Th-2 immune response with the multifaceted intensity of innate immunity. The current study examines plasma from people without HIV infection and with a low risk of HIV exposure for antibody capable of promoting HIV-induced IFN-α production to a degree that could explain how an otherwise, slow initially weak and virus-compromised IFN-α response could terminate HIV infection.

## Results

### Enhancement of HIV-induced IFN-a production by plasma from HIV-seronegative adults in geographic areas with high (Thailand) and low (USA) risks of HIV-infection

**Figure 1:**
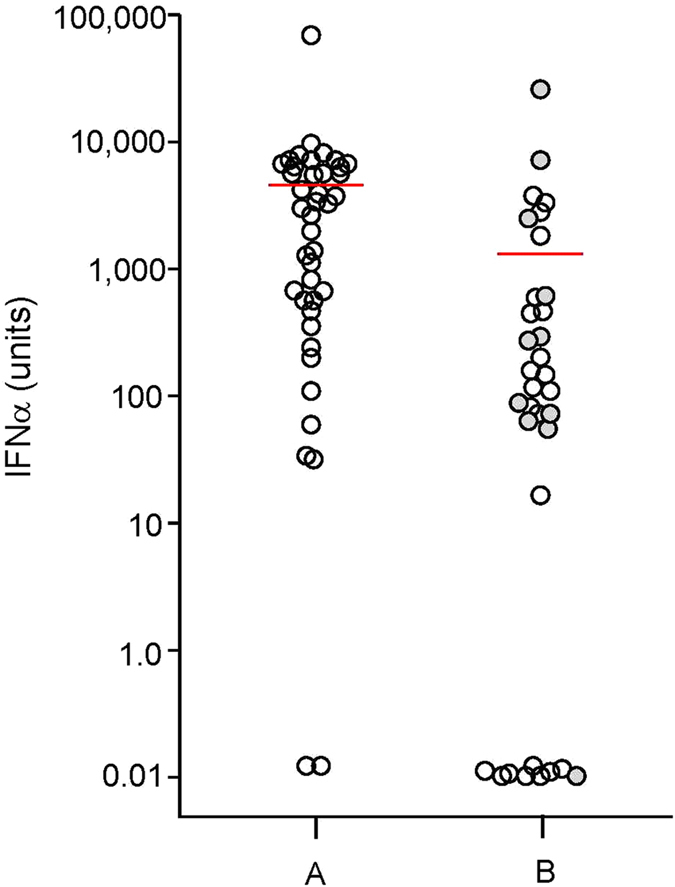
The ability of plasma from persons without HIV infection to promote HIV-induced IFN-α production. pDC IFN-α production induced by HIV plus plasma from: (column A) 43 HIV-seronegative Thai residents and (column B) 33 low risk USA residents (24 confirmed HIV seronegative-open circles; 9 healthy clinic personnel-closed circles). Each circle represents HIV-induced IFN-α production in the presence of plasma from a single individual assayed a minimum of three times. The mean IFN-α titer is indicated by the horizontal red line for each group (P < 0.001). Plasma did not induce IFN-α in the absence of HIV (data not shown).

Plasma from 41 of 43 reproducibly HIV-seronegative individuals living in a relatively high risk environment in Thailand promoted IFN-α production by pDCs exposed to limited numbers of virus particles in the range of an MOI of 0.001–0.01. Low virus concentrations were selected to simulate single transmitted founder viruses known to initiate mucosal infection in susceptible individuals^6^. HIV alone at these concentrations induced minimal IFN-α production in the range of 10–30 units. While in the presence of Thai seronegative plasma HIV induced IFN-α titers ranged from 33 to 67,252 units (average 4,585 units) of IFN-α (Fig. 1 column A).

Plasma from 24 of 33 individuals residing in a low risk area was also shown to enhance HIV-stimulated IFN-α production. No measurable IFN-α was detected in pDC cultures without virus or plasma, or in pDC cultures containing plasma without HIV (data not shown). Plasma from individuals residing in the USA induced IFN-α titers from 16 to 25,356 units with an average of 1,268 units (Fig. 1 column B). Plasma from 65 of 76 (86%) individuals from these two geographically and ethnically distinct populations promoted HIV-induced IFN-α production. The magnitude of enhancement was significantly greater for the Thai as compared to the USA population (P < 0.001).

### Effect of plasma on the rate and magnitude of HIV-induced IFN-α production

**Figure 2:**
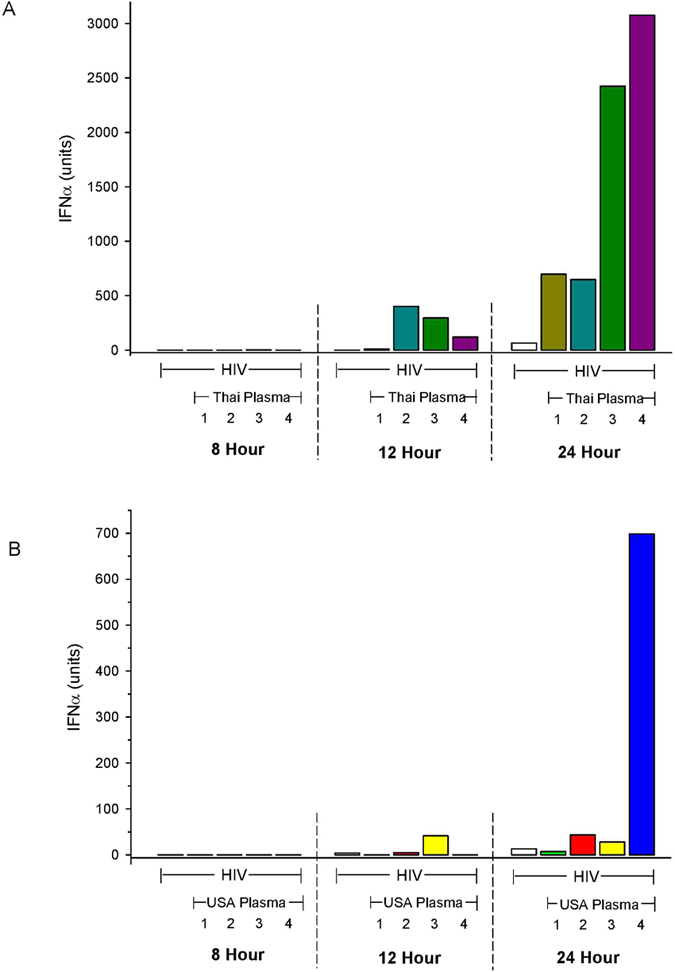
The time of appearance and titer of IFN-α induced by HIV in the presence or absence of HIV-seronegative plasma from geographic areas of high (**A**) and low (**B**) HIV prevalence. IFN-α titers were measured at 8, 12 and 24 hours. Bars represent the titer of IFN-α. Each plasma number represents a separate donor. Samples with no IFN-α titer are indicated with a single line.

Previously, we identified increased sensitivity to induction by low viral inoculums, increased rate and quantity of IFN-α production as defining characteristics of the process by which circulating IgG promotes the efficiency of IFN-α production^17^,^24^. The rate and magnitude of IFN-α production by pDC was examined at intervals in cultures containing Thai plasma and HIV or HIV alone (Fig. 2A). HIV alone first induced IFN-α with a titer of 65 units at 24 hours. In comparison IFN was detected as early as 8 hours in cultures containing HIV and Thai plasma, was present in 4 of 4 cultures with an average of 200 units at twelve hours and a titer of 650 to 3,050 units at 24 hours (Fig. 2A). The same pattern although with lower titers was noted for IFN-α production induced by HIV in the presence or absence of plasma from USA residents (Fig. 2B). Plasma from USA residents produced less striking acceleration of IFN-α production (Fig. 2B).

### Characterization of the plasma component that promotes HIV-induced IFN-α production

**Figure 3:**
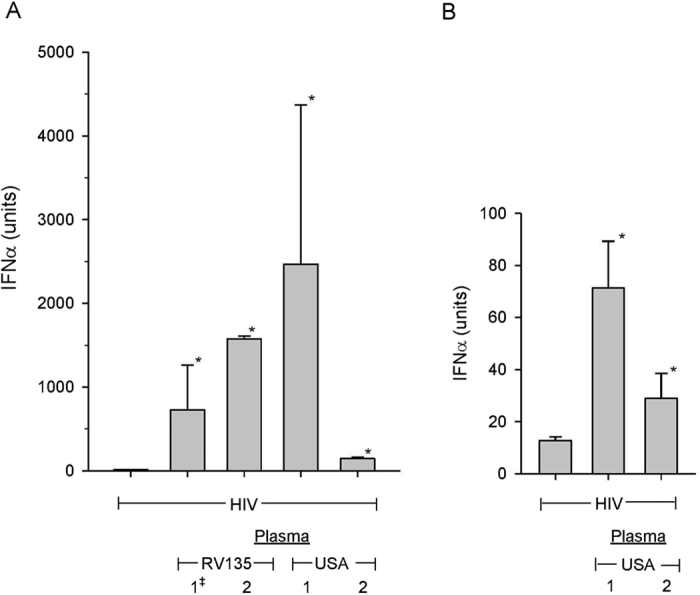
Promotion of HIV-induced IFN-α production by Protein G purified IgG. Protein G purified IgG was added at a final concentration of 70 μg/ml along with sub-stimulatory HIV concentration to pDC (**A**) or PBMC (**B**) cultures. Column fractions were assayed in triplicate (Bars). (*) denotes statistical significance (P < 0.03) between IFN-α titers induced by IgG and HIV compared to HIV alone. (**A**) (1^‡^) Pooled plasma samples from two Thai individuals or (2) from an individual donor. (**A,B**) USA plasma from individual donors.

Plasma derived, protein G purified IgG was examined for the ability to promote HIV-induced IFN-α production in pDC or PBMC cultures. When added to pDC cultures IgG derived from Thailand and USA plasma promoted IFN-α production to a variable, but statistically significant degree that ranged from a low of 155 to a high of 1,500 units while HIV alone induced an average IFN-α of 13.5 units (Fig. 3A). Similar results, but with lower IFN-α titers, were obtained when IgG derived from USA plasma were examined in PBMC cultures (Fig. 3B). Unbound column samples had no IFN-α promoting activity. IgG that promoted HIV IFN-α production was defined as IFN-α Promoting Antibody or IPA.

### Immunoglobulin G-HIV complex formation and the induction of IFN-α

**Figure 4:**
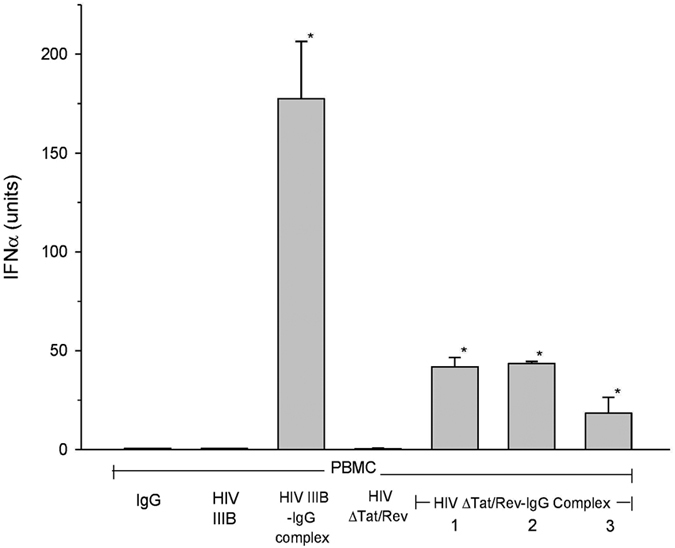
Production of IFN-α promoted by IgG-HIV complex. Protein G purified IgG bound to HIV IIIB or replication deficient HIV ΔTat/Rev promoted IFN-a production in PBMCs. HIV bound to IgG #1, 2, 3 indicates an individual USA plasma sample. IFN-α titer for HIV IIIB-IgG complex is an average of two experiments. Unbound HIV washed from the column and Protein G purified IgG alone is also presented. (*) denotes statistical significance (P < 0.03) between IFN-α titers induced by HIV-IgG complex compared to HIV alone.

Plasma with a demonstrated ability to promote HIV-induced IFN-α production was incubated with HIV to allow antibody-virus complex formation. Immunoglobulin G bound to HIV was captured on and eluted from either gravity or spin columns containing Protein G, back dialyzed and added to cultures at an average final concentration of 70 μg/ml. Eluted complexes were identified by their ability to promote IFN-α production in PBMC cultures (Fig. 4). HIV IIIB, and replication deficient HIV IIIB ΔTAT/Rev virus were used for antigen-antibody complex formation. Antibody-HIVIIIB complexes induced an average IFN-α titer of 175 units, and an average of 38 units for HIV ΔTAT/REV (Fig. 4). HIV not bound to IgG present in the incubation mixture passed through the column and did not induce IFN-α.

### Interferon characterization and assay selection

**Figure 5:**
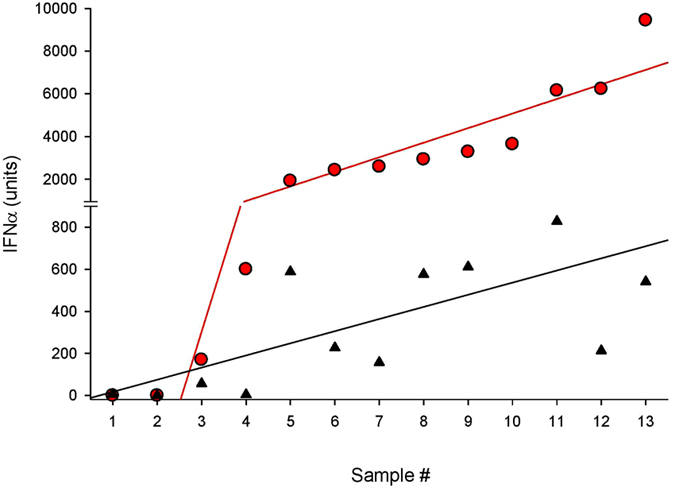
Comparison of IFN-α quantification by biological and immune (ELISA) assays. HIV-induced IFN-α produced in cultures were measure using a bioassay (red circles) and a human IFN-α multi-subtype immunoassay (black triangles). The red and black lines are a computer generated best fit curve. Samples contained: pDC (#1), a low HIV concentration (#2), and individual plasma (#3–13).

Immune-specific reactivity of IFN-α produced in pDC and PBMC cultures was examined by bioassay in A549 cells and by an IFN-α multi-subtype immunoassay. Both methodologies produced concordant results, with the bioassay reporting approximately 10-fold higher IFN-α titers (Fig. 5). Antiviral activity from pDCs and PBMCs induced by HIV in the presence of IPA from persons residing in the USA or Thailand were neutralized by >99% with sheep polyclonal antibody (Ab) to human IFN-α. No loss of antiviral activity occurred when IFN-α preparations were incubated with anti-IFNγ antibody (data not shown).

### Antibody-Mediated Enhancement of HIV Induced IFN-a Production Requires FcR Binding and Endosomal Processing

**Figure 6:**
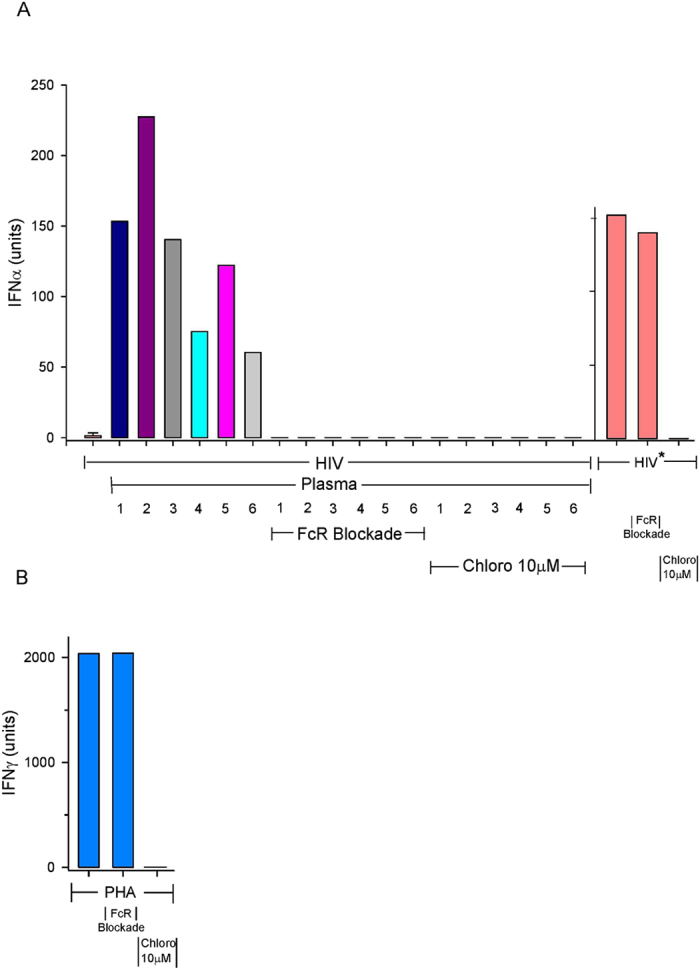
Effects of FcR Blockade and Chloroquine on IFN-α induced by HIV in the Presence of IPA. (**A**) Individual plasma was added along with a minimally stimulatory HIV concentration to either PBMC alone or PBMC pretreated with FcR Blocking Reagent or with 10 μM Chloroquine. Individual Thai and USA plasma are indicated by numbers 1, 2, 3 and 4, 5, 6 respectively. (*) denotes an IFN-α stimulatory concentration. (**B**) IFN-γ induced by PHA in either PBMC alone or pretreated with FcR Blocking Reagent or Chloroquine (10 μM). Samples with no IFN-α titer are represented by a single line.

IFN-α production promoted by Thai plasma (samples 1–3) and USA (samples 4–6) individuals were inhibited to undetectable levels in the presence of the endosomal alkalinizing agent chloroquine (Fig. 6). Additionally, IPA mediated HIV induced IFN-α production was reduced to undetectable levels when PBMCs were pre-incubated with an FcR blockade reagent containing a mixture of antibodies that block surface Fcγ receptors (Fig. 6). The combination of blocking antibodies had no effect on PBMC to produce the FcγR-independent induction of IFN-γ by phytohaemagglutinin (PHA), while chloroquine inhibited interferon induction consistent with the known dependency of interferon production on endosomal processing^25^,^26^. Figure 6 is representative of three separate experiments.

## Discussion

The current study is to our knowledge the first report of antibody in uninfected humans with the potential to protect against HIV infection. We demonstrate that the majority of persons without HIV-infection, and a low predictability of HIV exposure have IgG that increases the rate and quantity of HIV-induced IFN-α production. This naturally occurring interferon promoting antibody (IPA) enables pDC and PBMC to rapidly produce high IFN-α titers in response to HIV concentrations which independently stimulate little or no IFN-α production. IFN-α produced *in vitro* by a few cells and rapidly diluted in a disproportionately large volume of cell culture media, underestimates the intensity of IFN-α production in the restricted confines of the mucosa where concentrations of IFN-α in millions of units can accumulate in the immediate vicinity of HIV exposed pDC^27^. IFN-α concentrations of this magnitude can create an intense focused multifaceted anti-HIV response capable of preventing the infection of cells in the vicinity of the initial HIV-pDC interaction^27^,^28^,^29^,^30^,^31^,^32^. Naturally occurring IPA confers immune specificity to a non-specific but powerful innate immune response which affords the potential to extinguish viral replication before the creation of cells with permanent latent infection^2^,^5^,^33^,^34^.

HIV-specific neutralizing and non-neutralizing antibodies (non-Abs) have been derived from plasma in selected individuals with long-standing infection^17^,^35^,^36^,^37^,^38^. Broadly neutralizing antibodies (bnAbs) have been demonstrated to inhibit HIV replication in monkeys and humanized mice when administered before or concurrently with HIV^39^,^40^,^41^,^42^,^43^. The Fcγ portion of bnAbs and non-nAbs antibodies are crucial in creating a potent host defense mechanism against infection that include ADCC, ADCVI, phagocytosing antibody and immune complexes that block CD4+ T cell recruitment^39^,^44^,^45^,^46^,^47^,^48^,^49^,^50^,^51^,^52^. Our current study adds IPA, an antibody with the potential of creating robust production of IFN-α as a mediator of an Fc-dependent antiviral state capable of preventing HIV infection. To our knowledge, IPA is the only Fc-dependent antibody that does not require preexisting HIV-infection to exert its antiviral effects^35^,^36^,^37^,^38^. The wide range of IPA concentrations present in different individuals raises the possibility that low or absent IPA would not provide a barrier to HIV replication while high IPA activity would. Variation in IPA concentrations between individuals shown in the current studies may be explain person-to-person differences in IFN-α sensitivity reported for founder viruses isolates^5^,^52^,^53^,^54^.

The presence of HIV-IPA in a large proportion of the general population in non-endemic areas is supported by reports of HIV specific memory CD4+ T-cells and anti-HIV antibody of unknown function in HIV seronegative people also residing in the San Francisco Bay Area in the USA^55^,^56^,^57^. The nearly universal prevalence of IPA in our subjects contrasts with their low probability of HIV exposure. Plasma donors in the USA live in a low risk environment, while Thai subjects were selected on the basis of low personal risk profiles and documentation of persistently HIV-seronegative tests [AP-VaxGen Protocol v1.12, Jan. 18, 2001]. The IPA titers in both populations ranged from undetectable levels to tens of thousands of units which as noted above is consistent with broad variations in transmitted founder virus IFN-α resistance^5^,^53^,^58^. Possible IPA origins include exposure to unrelated viruses, sensitization to cross reacting antigens, auto-antibodies, low affinity polyreactive antibody and naturally occurring germ line IgG^59^,^60^,^61^,^62^. Regardless of its origins, IPA offers a potential explanation for the means by which IFN-α can provide a barrier to primary HIV infection^7^,^8^,^9^,^63^. Confirmation of naturally occurring IPA as a correlate of protection has the potential to expand an understanding of the pathogenesis of HIV infection and to offer alternatives for vaccine development.

## Materials and Methods

### Blood Donors

Blood was collected by venipuncture from healthy volunteers living in the San Francisco Bay Area of Northern California. Blood was drawn into 4.5 ml tubes containing lithium heparin (Becton Dickenson, Franklin Lakes, NJ) and processed the same day for isolation of pDC, PBMC or plasma. All individuals signed an informed consent. This study was approved by the Institutional Review Board for the Department of Veterans Affairs Northern California Health Care System. All methods were performed in accordance with the relevant guidelines and regulations.

### Plasma

Plasma was obtained from 3 sources: (1) Healthy HIV seronegative volunteers as noted above (2) Plasma samples from clinic attendees undergoing a *Centers for Disease Control and Prevention* (CDC) recommended screening for HIV infection using the HIV Ag/Ab Combo assay (Abbott Laboratories, Abbott Park, IL). De-identified HIV seronegative plasma specimens were provided by the clinical laboratory immediately prior to being discarded. Samples were numbered sequentially and stored at −80 °C in the research laboratory and (3) plasma samples from subjects taken prior to participating in a Phase II HIV vaccine trial (RV135) in Thailand kindly provided by Dr. Jerome Kim of the U.S. Military HIV Research Program. Subjects were selected for participation based on low personal risk profiles despite residing in a high risk environment. A low risk designation was supported by the fact that all RV135 samples were HIV-Serology negative at the time they were obtained and when examined intermittently during the next 12 months [AP-VaxGen Protocol v1.12, Jan. 18, 2001].

### Peripheral blood mononuclear cell (PBMC) cultures

Venous blood was obtained from healthy HIV seronegative volunteers as noted above. Isolation and preparation of PBMC was performed as described previously^64^. PBMC were isolated on Histopaque (Sigma Aldrich, St. Louis, MO), and suspended at a final concentration of 2.0 × 10^6^ cells/ml in RPMI-1640 (Sigma Aldrich, St. Louis, MO) with 10% fetal bovine serum (FBS) (Hyclone, Logan, UT) and gentamicin (10 μl/ml). PBMC suspensions were dispensed in 400 μl quantities into loosely capped disposable borosilicate glass tubes (16 × 100 mm) and maintained overnight at 37 °C in a humidified 5% CO_2_ atmosphere incubator.

### Plasmacytoid dendritic cell (pDC) cultures

A Plasmacytoid Dendritic Cell Isolation Kit II (Miltenyi Biotech, Auburn, CA) was used to magnetically separate unlabeled pDCs from PBMCs. pDC isolation was performed according to the manufacturer’s instructions and yielded a pDC purity >95%. Purified pDCs were suspended at 2.0 × 10^4^ cells (200 μl) in RPMI-1640 with 10% FBS and gentamicin (10 μl/ml). pDC suspensions were dispensed into a 96-well cell culture plates (Santa Cruz Biotechnology Inc., Dallas, TX) and maintained in a humidified 5% CO_2_ atmosphere incubator at 37 °C for 24–48 hours.

### Demographics

#### Thailand Plasma Samples

The total study population consists of 43 healthy HIV-seronegative Thai adults, approximately equal number of males and female between the ages of 20–50 years old. Subjects were participants in the phase II (RV135) precursor to the phase III RV144 vaccine trial, certified that they avoided high risk behavior for HIV acquisition, and had initial and periodic negative HIV-Western Blot tests during the 12 month study.

#### USA Plasma, pDC and PBMC Samples

Both healthy male and female clinic personnel in a 2:1 ratio donated blood for pDC and PBMC isolation. All but three experiments used pDC from males. HIV-seronegative plasma were obtained from individuals residing in the USA at a clinic for military veterans where the majority of patients are male.

### Viruses

HIV-1 (strain IIIB) concentrated 1000 times from infected H9 cell culture supernatant (10^7^ TCID/ml), and HIV-1 (strain IIIB) propagated in H9 cells and gradient purified from culture supernatants (10^6.8^ TCID/ml) were purchased from Advanced Biotechnologies Inc. (Eldersburg, MD) and used interchangeably in experiments. Stock virus was stored in aliquots (60 μl) at −80 °C. Each aliquot was rapidly defrosted at room temperature and refrozen a maximum of ten times. HIV-1 MC99IIIBΔTat-Rev and CEM-TART cells were acquired through the NIH AIDS Reagent Program, from Drs. Herbert Chen, Terence Boyle, Michael Malim, Bryan Cullen, and H. Kim Lyerly (Germantown, MD). HIV-1 MC99IIIBΔTat-Rev was propagated in CEM-TART cells as previously described by Chen *et al*.^65^. Cell culture media were collected on day 15, centrifuged (3000 rpm) at 4 °C, divided into aliquots (1 ml) and stored at − 80 °C. Encephalomycocarditis (EMC) virus used in the IFN-α bioassay was prepared as described previously^66^.

### Interferon Alpha Production

After PBMC or pDC were isolated and dispensed, either 1000x pelleted HIV-1 IIIB with a TCID_50_ titer of 10^7.5^ TCID_50_/ml was added at an MOI of 0.001–0.01 or gradient purified HIV-1 IIIB with a TCID_50_ titer 10^6.8^ TCID_50_/ml were used interchangeably and were added to cultures at an MOI 0.001–0.05. A final dilution of human plasma (1:100) or purified IgG (1:10) were added to sample cultures. MOI for each HIV-1 virus type was selected based on its ability to induce minimal (<100U) IFN-α production^17^,^18^. HIV and plasma or IgG were added to the cultures without pre-incubation and gently agitated.

### Interferon Alpha Bio-Assay

Interferon alpha titer was reported as units (U) and quantified as a reciprocal of the dilution producing a 50% photometric CPE reduction produced by EMC virus in the continuous human A549 cell line (American Type Culture Collection, Manassas, VA)^66^. HIV did not directly stimulate development of an anti-viral state in A549 cells. When present, HIV-induced IFN-α titers in cultures containing virus alone were subtracted from IFN-α titers produced in the presence of plasma.

### Interferon Alpha Enzyme Linked Immunosorbent Assay (ELISA)

Interferon alpha from stored culture samples used in bioassays were measured using an ELISA kit (PBL Interferon Source, Piscataway, NY) and performed according to the manufacturer’s protocol. The assay is specific for detecting IFN-α in human culture media with an extended sensitivity range of 156–5000 pg/ml, and does not recognize IFN-β or IFN-γ. *ELISA generated IFN-α titers were converted from picograms to units using the conversion formula of 3 pg/ml to 1 unit of IFN-α.*

### Interferon Alpha Neutralization Assay

Neutralization of IFN by mono-specific antisera was performed as previously described^64^. In brief, 10 μl volumes of human IFN-α or IFN-γ antisera were diluted to a concentration of 500–1000 neutralizing units and incubated for 1 hour at 37 °C with diluted specimen (90 μl) containing approximately 30U of IFN-α. Serial 2-fold dilutions were made of the antiserum IFN mixtures and assayed for antiviral activity.

### Inactivation of IFN-α Production by Chloroquine or FcR Blocking Reagent

#### PBMC cultures with HIV or HIV and serum contained either

*Chloroquine* (Sigma Aldrich, St. Louis, MO) was added at a final concentration of 10 μM. This concentration was shown by others to maximally inhibit endosomal acidification while maintaining PBMC viability^67^. Chloroquine remained in PBMC cultures for the duration of the assay.

*FcR Blocking Reagent* used in accordance with the manufacturer’s protocol (Miltenyi Biotech, Auburn, CA). PBMC and FcR blocking reagent were co-incubated at 4 °C for 15 minutes, unattached blocking reagent was removed by sequential centrifugation prior to resuspending PBMC in culture medium.

Control cultures containing 5 μg/ml phytohaemagglutinin (PHA) (Sigma Aldrich, St. Louis, MO) or PHA and serum were incubated for 48 hours.

### *Streptococcus Protein G* Isolation and Recovery of Purified IgG

Immunoglobulin G was purified on a Protein G column from individual plasma from USA and Thailand. Thai plasma available in limited quantities was conserved by combining two samples to have sufficient volume for purification while preserving the remaining plasma for future studies. Human plasma was diluted 1:1 with PBS (1 M) at pH 7.25 and clarified by micro-centrifugation. One milliliter was added to a 3 ml column containing Protein G-Agarose Fast Flow beads (1 ml) (Sigma Aldrich, St. Louis, MO) equilibrated with PBS (1 M) pH 7.25 buffer, washed with PBS (30 ml, 1 M) pH 7.25 and eluted with trimethylamine (10 ml, 100 mM) at pH 10.5 into NaH_2_PO_4_ (60 μl, 1 M) at pH 4.5 (final pH 7.0), back dialyzed against RPMI-1640 and immediately added to either PBMC or pDC cultures.

### HIV Bound Human IgG Immune Complex Preparation

Human plasma was clarified by micro-centrifugation prior to complex formation. Buffers and elution procedures were the same as those used in IgG recovery described above.

#### Gravity columns

To reduce the risk of laboratory acquired infection, replication deficient HIV-1 MC99IIIBΔTat-Rev (50 μl) was used. HIV IIIB ΔTat-Rev was added to individual human plasma (0.5 ml), incubated for 1 hour at 4 °C, and diluted 1:1 with PBS (1 M) at pH 7.25. Replication deficient HIV-1 bound to IgG was isolated on Protein G-Agarose Fast Flow columns, collected in 0.5 ml volumes and eluted as described above.

*Spin columns* were used to isolate IgG-bound replication competent virus while reducing the infectious potential of gravity columns. HIV-1 IIIB (2.5 μl) in RPMI-1640 (400 μl) was incubated with plasma (400 μl) at 4 °C for 30 mins and added to Multipurpose Mini Spin Columns (BioVison Inc., Milpitas, CA) containing Protein G-Agarose Fast Flow beads (400 μl). Beads were washed 5 times with PBS (1 M) at pH 7.25 and 2–5 second pulse spins. HIV-1 bound IgG was eluted into tubes containing NaH_2_PO_4_ (50 μl, 1 M) at pH 4.5, and dialyzed overnight at 4 °C in RPMI-1640.

### Approval of experimental protocols

Experimental protocols were approved by the Institutional Review Board for the Department of Veterans Affairs Northern California Health Care System and all experimental protocols were performed in accordance with relevant guidelines and regulations.

## Additional Information

**How to cite this article**: Lum, T. and Green, J. A. Naturally Occurring Fc-Dependent Antibody From HIV-Seronegative Individuals Promotes HIV-Induced IFN-α Production. *Sci. Rep.* **6**, 37493; doi: 10.1038/srep37493 (2016).

**Publisher's note:** Springer Nature remains neutral with regard to jurisdictional claims in published maps and institutional affiliations.
